# Child-focused mHealth apps used in collaborative, real-world interventions: A scoping review

**DOI:** 10.1177/20552076261490207

**Published:** 2026-09-18

**Authors:** Simrin Pardal, Robin O’Hagan, Emmanuel Abaya-Kotey, Jean Anne Schnittker, Danielle Glista

**Affiliations:** 1 The National Centre for Audiology, 6221 Western University, London, ON, Canada; 2 School of Rehabilitation Therapy, Queen’s University, Kingston, ON, Canada; 3 School of Health Studies, Faculty of Health Sciences, 6221 Western University, London, ON, Canada; 4 School of Communication Sciences and Disorders, Faculty of Health Sciences, 6221 Western University, London, ON, Canada; 5 Department of Health and Rehabilitation Sciences, Faculty of Health Sciences, 6221 Western University, London, ON, Canada; 6 87724 Sonova AG, Zurich, Stäfa, Switzerland

**Keywords:** mHealth applications, pediatric rehabilitation, collaborative care, scoping review

## Abstract

**Introduction:**

The field of rehabilitation is experiencing an influx of mobile health (mHealth) applications designed to improve access to care and support adherence to therapeutic interventions. Rehabilitation encompasses a range of disciplines, including audiology, speech language pathology, clinical psychology, occupational and physical therapy, orthotics and prosthetics, and physical and rehabilitation medicine; each contributing to optimizing function and reducing disability for individuals with diverse health conditions. This scoping review aimed to synthesize the literature on pediatric-focused mHealth apps in real-world intervention studies led by rehabilitation healthcare professionals as part of a collaborative care practice.

**Methods:**

A comprehensive search was conducted using MEDLINE(R) ALL (Ovid), Embase and Embase Classic (Ovid), Nursing and Allied Health, Web of Science, Scopus, and CINAHL (EBSCO) databases. A published protocol guided study selection and data extraction based on defined objectives and inclusion criteria.

**Results:**

Forty-five peer-reviewed articles met the inclusion criteria. The studies addressed pediatric health conditions related to speech and language, hearing, mental health, physical functioning, autism spectrum disorder, and developmental disorders. Identified mHealth apps incorporated both active (e.g., two-way communication, tailored interventions, data uploads, interactive prompts) and passive features (e.g., reminders, digital diaries, Bluetooth connectivity, one-way messaging).

**Conclusion:**

This review demonstrates the emerging use of pediatric mHealth applications within collaborative rehabilitative models. Findings underscore the need for greater integration of mHealth tools into interprofessional pediatric rehabilitation and for the use of consistent evaluation frameworks to support equitable and evidence-based digital care.

## Introduction

According to a recent Survey on Children and Youth, mobile device use among youth (aged 12-17) is highly common and widespread, primarily used for purposes related to social networking, video/instant messaging, and online gaming.^1^ Many healthcare fields have tapped into the potential that the prevalence of mobile device ownership offers. Included in this are health programs, developed for the delivery of health-related information, resources and services through mobile means.^2^ Mobile health (mHealth) involves the use of mobile phones, personal digital assistants, patient monitoring devices, and other wireless devices, in the context of medical and public health practice.^3^ Within healthcare fields, including rehabilitation, there is a large influx of mHealth applications designed to improve access and adherence to care. Rehabilitation incorporates interventions optimizing function and reducing disability for individuals living with one or more health conditions, and encompasses audiology and speech-language pathology, clinical psychology, occupational and physical therapy, orthotics and prosthetics, and physical and rehabilitation medicine.^4^

When providing rehabilitative care, a clinician should focus on the use of patient-centred care approaches, to encourage active collaboration and shared therapeutic decision making between the patient and provider, known as a collaborative care model.^5^ mHealth can be integrated into collaborative care models, where collaborative care practice is a desired and necessary component of health care delivery, allowing quality patient-centred care with shared decision making.^6,7^ This collaborative care model relies on patient and/or family, and provider engagement in order to understand the implications of mHealth on patient outcomes.^8^ To improve accessibility and uptake of rehabilitation services, professionals may integrate mHealth applications (or apps) into clinical care processes. mHealth apps accompanying rehabilitation services can act as an interface between a patient and a provider and between the patient and their care process.^9^ Previously cited benefits of mHealth include real-time data collection, intervention in natural environments, lower costs, and health behaviour tracking with feedback.^10^ Additionally, mHealth can improve access to care by reducing geographical barriers such as travelling to medical centres, which is beneficial for those living in rural locations, those who rely on public transit, and those with lower socioeconomic status (SES).^11^ Apps used within rehabilitation services offer the potential to improve patient-centred care within a collaborative care model; a systematic review by Florian Ross on smartphone apps in hearing healthcare found that engaging patients in hearing aid fitting via smartphone apps led to better hearing aid outcomes.^12^

The large majority of adolescents own or use a mobile device daily, with a global estimation of 95% of adolescents reporting that they have access to cell phones.^13^ mHealth has the potential to improve access and engagement in health services for adolescents, as it can offer an adjunct or alternative to in-person treatment, thereby supporting the rehabilitative care process.^14–16^ Current literature has only explored mHealth apps-based interventions with children in the context of physical activity or children living with specific physical conditions, such as peritoneal dialysis.^17,18^ Although emerging in the adult literature, rehabilitation-based mHealth apps have yet to be explored extensively with pediatrics in real-world environments. Evaluating care within “real-world” environments provides the opportunity for researchers to evaluate health outcomes and/or performance outside of lab-based or simulated environments, in a more naturalistic, information-rich way. Real-world data refers to a patient’s health status or the delivery of health care across a variety of sources, including data captured by digital health technology during the treatment and management of the patient^19^; this type of data is often used to generate clinical recommendations and support decision-making.

mHealth apps have many different components, ranging from gamification of different features, chat functionality, and real-time support. These app components, or features, can be categorized into two different domains: interactive or passive.^20^ *Interactive components* encompass features that the end-user must actively and purposely engage with, including two-way or tailored SMS or messaging, completing a task as part of the action/treatment plan or personalized health goals, uploading biometric data to their care provider for review and real-time feedback, interactive prompts requiring end-user interaction, and a clinical decision support system to assist end-users with self-management. *Passive features* do not require the end user to actively engage with the app, as they often run in the background; these features include reminders, electronic diaries (automatic storing and display of biometric measurements and app use), Bluetooth connections, and one-way messaging.

Our team conducted a scoping review of the current literature to evaluate mHealth apps within pediatric rehabilitation services, used in real-world scenarios, and within collaborative models of care. This scoping review explored the barriers and facilitators to pediatric patient engagement with mHealth apps and providers’ readiness to implement mHealth apps into practice (including engagement readiness). For this review, “collaborative care” will include interventions co-led by healthcare professionals within rehabilitation sciences and include direct pediatric patient involvement in manipulating or interacting with the app as part of the intervention. This study aimed to gather, synthesize, and summarize pediatric-focused mHealth apps that have been used in real-world intervention studies by healthcare professionals within rehabilitation sciences, as part of a collaborative care practice, following the Population-Concept-Context (PCC), as outlined in Table 1^21^ This included the collection of evidence around mHealth quality, safety, and user engagement; participation roles; patient populations, type of intervention, targeted childhood health conditions; mHealth app objectives; barriers and facilitators to user engagement; contexts within which app(s) are being used; reported training regimes; and reported outcomes of app use in rehabilitation therapy.

**Table 1.** PCC structure.

Component	Description
Population	Pediatric clients (individuals under 18 years of age) who have received some form of care from a rehabilitation healthcare professional as part of an intervention.
Concept	The use of mHealth apps in a collaborative care practice, involving interventions co-led by healthcare professionals within rehabilitation sciences and includes direct patient (child) involvement/engagement with the app as part of an intervention (e.g., self-monitoring, intervention adjustment).
Context	Real-world app use scenarios, as part of rehabilitative care, with varying delivery models. No limitations on geographic location, racial or ethnic backgrounds, gender or app development phase.

## Methods

This scoping review was conducted following the Joanna Briggs Institute (JBI) methodology for scoping reviews and reported according to the Preferred Reporting Items for Systematic reviews and Meta-Analyses (PRISMA) extension for Scoping Reviews (Appendix A).^22^ In alignment with research ethics policies, scoping review projects are exempt from the research ethics review processes based on the use of secondary and anonymized information.^23^

### Protocol and registration

Prior to initiating the full literature review, a preliminary search using MEDLINE and Embase was conducted to determine whether there were any published systematic or scoping reviews providing synthesized data on children’s use of mHealth apps within allied health fields. No published reviews were discovered following the preliminary search. Upon completion of the preliminary search, the research team developed a scoping review protocol, outlining the objectives, inclusion criteria, and methods for this scoping review, and published the protocol through the Open Science Framework (OSF).^24^

### Eligibility criteria

This scoping review included articles that were: 1) published and/or accessible in the English language, 2) accessible electronically, 3) peer-reviewed, 4) available in full-text, 5) contained original research findings, 6) described a care context including the use of mHealth apps, 7) guided real-world treatment/intervention, 8) care provision beyond assessment and/or screening only, 9) care included pediatric populations (0-17 years, inclusive), and 10) mHealth app use was co-led by a healthcare professional within rehabilitation sciences with direct patient (child) manipulation of the app as part of the intervention. There were no limits placed on the date range of articles included in this review. Original research papers, including primary research studies, case studies, and pilot studies were included in this scoping review. The following sources of evidence were excluded from this review: text and opinion papers, grey literature (non-peer-reviewed), scoping or systematic reviews, and research protocols. Additionally, the research team conducted handpicking, evaluating the reference lists of all included sources of evidence to identify additional articles for inclusion.

### Information sources

Six databases were used to obtain articles for this review: MEDLINE(R) ALL (Ovid), Embase and Embase Classic (Ovid), Nursing and Allied Health, Web of Science, Scopus, and CINAHL (EBSCO). Database searches were completed on November 25, 2024.

### Search strategy

A Teaching and Learning Librarian (University of Western Ontario) assisted in refining the search strategy. An initial search strategy was created using the MEDLINE(R) ALL (Ovid) and Embase Classic (Ovid) databases. The search strategy was first created for MEDLINE(R) All (Ovid) database and modified for each additional database. The final search strategy was based on and modified from the search terms outlined in Table 2. The search strategy, including all identified keywords and index terms, was adapted for each included database and/or information source (Appendix B). Field codes were used to indicate title or abstract (e.g., tw.), word in author-provided keywords (e.g., kf.), along with truncation symbols to search alternate endings for words (e.g., *).

**Table 2.** MEDLINE search terms.

Category	Search terms
Population of interest	Pediatrics (pediatric* or paediatric*), Child (child*, Child, Preschool), Adolescent (adolescent*, teen*)
Delivery modality	Mobile Applications (mobile app*), Telemedicine, mHealth* (m-Health*, mobile health*), mobile treatment*, mobile intervention*, smartphone app*, connected health*
Rehabilitation field	Audiology (Audiologists, audiolog*), Speech-Language Pathology (speech or language therap* or patholog*), communication sciences and disorders (communication disorder*), Occupational Therapy (occupational therap*), Physical Therapy (physical therap*, physiotherap*), Prosthet*, orthot*, Psychiatric Rehabilitation, Physical and Rehabilitation Medicine (Rehabilitation Nursing or Rehabilitation, physical medicine doctor*, rehab* doctor* or nurse*), Clinical Psychology (clinical psychol*), rehab* professional* (science professional*, science*), rehabilitation, activities of daily living, animal assisted therapy, art therapy, bibliotherapy, cardiac rehabilitation, correction of hearing impairment, dance therapy, early ambulation, exercise therapy, music therapy, neurological rehabilitation, recreation therapy, rehabilitation of speech and language disorders, vocational rehabilitation, telerehabilitation

*Note.* Search terms were used for all databases. For MEDLINE and CINAHL subject headings, official words, and/or phrases selected to represent concepts (e.g., audiology) were also searched. tw. title or abstract; kf. word in author-provided keywords.

*Alternate endings for words.

### Evidence selection

The scoping review was conducted using the Covidence platform^25^ and followed the JBI’s four stages of review: Stage 1: import, Stage 2: title and abstract screening, Stage 3: full-text review, and Stage 4: data extraction. Prior to the title and abstract screening, an initial calibration exercise was conducted to ensure that inclusion criteria were consistently applied by all members of the research team.^26^ This calibration exercise was completed by the research team during two team meetings (DG, SP, RO, and DG, RO) using the first 10 articles in Covidence, sorted by “most relevant”. The lead reviewer (SP), along with one additional reviewer (DG, RO, JR, or EA), independently assessed all articles at each phase against the inclusion criteria, with potential articles included for the next stage using the tags “yes” or “maybe”. Throughout each stage of the review, conflicts were resolved by a moderator (DG) or through team discussion (SP, RO, JR, EA). Conflicts most often arose surrounding what care could be considered within rehabilitation sciences.

### Data extraction

Following the screening phases, the team completed data extraction using the modified data extraction tool within the Covidence software. The data extraction tool was modified according to the pre-determined extraction form created by the team prior to initiating the scoping review phases. The research team completed a calibration exercise with the first article to ensure consistency in extracted data. As with the screening step, the lead reviewer (SP) along with one other reviewer (DG, RO, JR, or EAK) independently extracted information from each article. Data items are outlined in Appendix C.

### Evidence level

To summarize the various types of studies included in this scoping review, the research team followed the Johns Hopkins evidence level and quality guide, categorizing each study’s research design and distinctive features.^26,27^ The evidence levels vary from 1- experimental: randomized controlled trials; 2- quasi-experimental: non-equivalent control groups, single test, time series, untreated control with repeated measures, single subject repeated treatment, and crossover designs; 3- nonexperimental (quantitative): descriptive, predictive, explanatory, or time-dimensional studies; 4- qualitative: basic qualitative descriptive, ethnography, grounded theory, phenomenology, narrative inquiry, and case studies; and 5- mixed methods: convergent parallel, explanatory sequential, exploratory sequential, and multiphasic studies.^27,28^

### Data synthesis

Extracted data was collated in Covidence by RO prior to data analyses, where independent reviewer data was copied into each cell if found missing from the second reviewer extractions. The data was then exported as an Excel (.csv) file from Covidence allowing for data analyses.

### Selection of sources of evidence

A total of 4686 articles were identified for screening, and an additional 1460 duplicates were removed using Covidence. One article was handpicked at the full-text review stage. Forty-five articles were included for data extraction, as depicted in the PRISMA-ScR flow diagram (Figure 1). Articles were excluded at the full-text screening stage for the following rationale: 1) care was not within rehabilitation sciences; 2) contained only adult populations; 3) full-text not available; 4) care was not provided in real-world scenarios; 5) no mention of co-led app use; 6) no mobile app used by a child; 7) care was exclusive to assessments, screening, or monitoring; 8) care was not related to a child with a disability; 9) care was not considered an intervention; 10) study was not written or accessible in the English language; 11) study was grey literature, review, or opinion article, not containing original data; and 12) did not contain a description of the study population.

**Figure 1.**
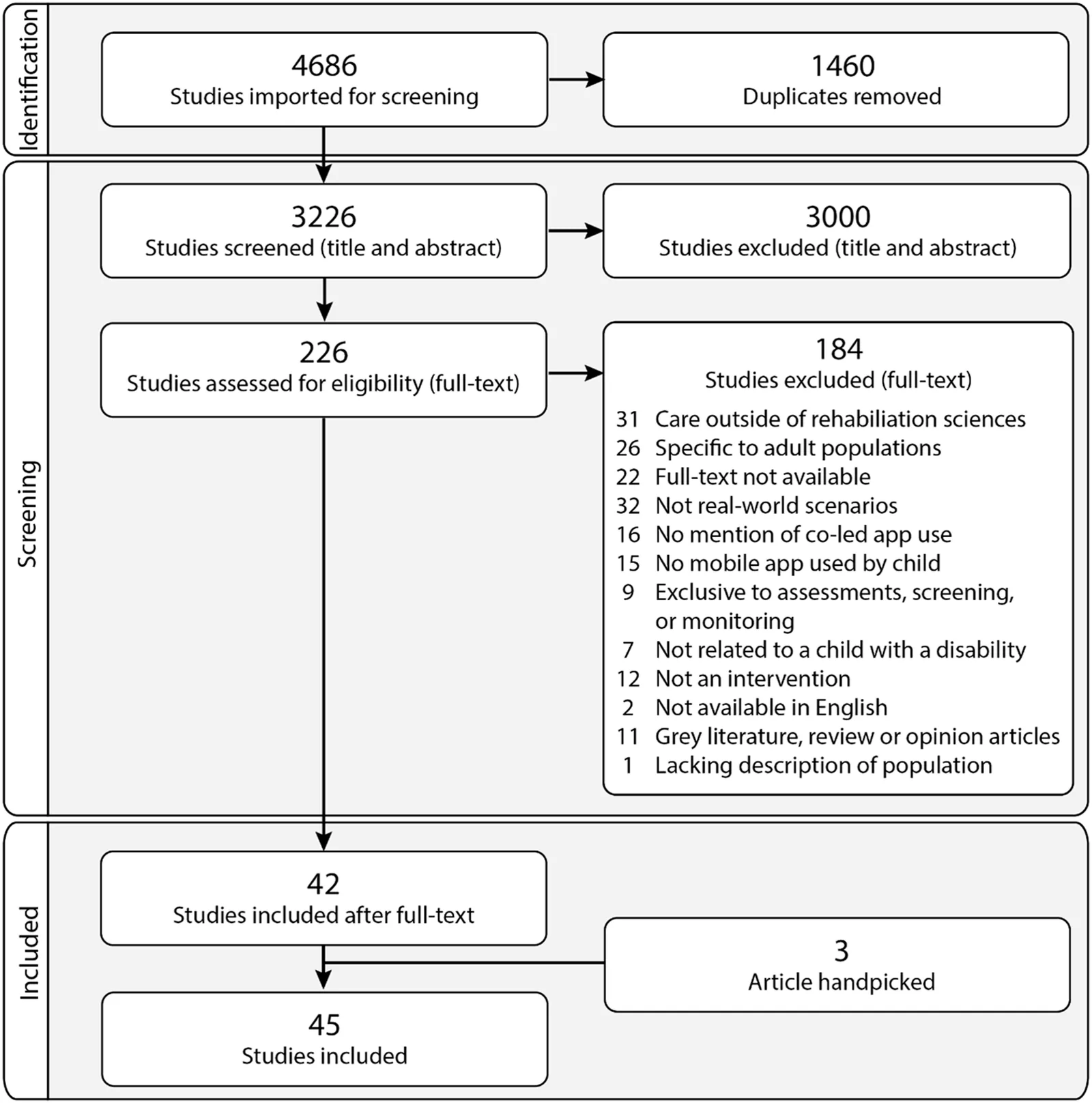
PRISMA-ScR flow diagram.

## Results

### Characteristics of sources of evidence

A summary of included articles is provided in Appendix D, including each study’s continent of origin; the level of evidence; the type of app assessment completed (if applicable); a summary of rehabilitation professionals involved in the care provided as part of the study; the total number of participating children included in each study and their related health condition(s); and the ages of the participants according to sited age ranges (or reported mean), in years. A total of 28 studies originated from North America, 11 from Europe, eight from Asia, and nine from Oceania; many studies included more than one country of origin. Table 3 provides a summary of all included studies and app-related components, such as operating system compatibility, the stage of development and source of the app, the type of app interaction experienced by the child, barriers and facilitators to app engagement, and related engagement outcomes. The included studies also varied by the operating systems used, with the largest number supporting only android (n = 20, 44.4%), followed by only iPhone (iOS; n = 13, 28.9%), fewer supported both android and iOS (n = 4, 8.9%), iOS and mac operating system (OS X; n = 1, 2.2%), and one study supported iOS for study-provided devices but did not report for participant-owned devices (2.2%), and six studies did not report on the operating system used (13.3%).

**Table 3.** Summary of app components and related engagement moderators/outcomes.

Citation	Source/development	App classification	Type of interaction^1^		Engagement moderators		Engagement outcomes
			Interactive	Passive	Barriers	Facilitators	
Ahmed et al.^29^	Study-developed, prototype	Treatment/support	Prompts; treatment plan; upload data	None	Gamification (difficulty level); technological difficulties	Gamification	Not reported
Babb et al.^30^	Study-developed, pilot testing	Treatment/support	Treatment plan	None	Not reported	Not reported	Not reported
Badesha et al.^31^	Publicly available	Treatment/support; health promotion/prevention	Treatment plan	None	Inconvenience; familiarity/novelty; cultural acceptance	Not reported	Module completion may be greater for guided vs unguided intervention
Bent et al.^32^	Publicly available, pilot testing	Treatment/support; information sharing/communication	Treatment plan; upload data	One-way SMS and Messaging; Reminder	Not reported	Not reported	Not reported
Brouwer et al.^33^	Publicly available	Treatment/support	Treatment plan	None	Not reported	Not reported	Not reported
Bunnell et al.^34^	Publicly available	Treatment/support	Prompts; treatment plan; upload data	None	Not reported	Not reported	Not reported
Caron et al.^35^	Publicly available	Treatment/support	Treatment plan	None	Not reported	Various communication modalities; motivation	Not reported
Carson^36^	Publicly available	Treatment/support	Treatment plan	None	Not reported	Not reported	Not reported
Chan et al.^37^	Study-developed, pilot testing	Treatment/support	Treatment plan; upload data	Bluetooth technology	Developmental	Accessibility features	Not reported
Chung K. & Chung E.^38^	Study-developed, pilot testing	Treatment/support	Treatment plan; upload data	None	Developmental	Motivation; accessibility features; access to use	Facilitated engagement; reduced parental burden
Coutinho et al.^39^	Publicly available	Treatment/support	Treatment plan	None	Not reported	Previous experiences with apps/devices	More engagement, eagerness, and focus on tasks; sustained/longer attention
De-Jongh González et al.^40^	Publicly available	Treatment/support; health promotion/prevention; information sharing/communication	Prompts; treatment plan; two-way messaging	One-way messaging;	Not reported	Parental engagement; gamification	Not reported
de la Vega et al.^41^	Study-developed, pilot testing	Treatment/support; information sharing/communication	Prompts; treatment plan; upload data	Electronic diary; reminders	Technological difficulties	Notifications; ease of use; feedback	Not reported
Díez-Juan et al.^42^	Publicly available	Treatment/support	Treatment plan; upload data	Electronic diary	Not reported	Not reported	Not reported
Gačnik et al.^43^	Study-developed, pilot testing	Treatment/support	Prompts; treatment plan	None	Not reported	Engaging graphics; ease of use; accessibility features; motivation	Enjoyment with tablet exercises over traditional tools; motivated to practice longer; preference for tablet-based exercise over traditional
Gazibegovic et al.^44^	Publicly available	Treatment/support	Treatment plan	Bluetooth technology	Hearing aid connectivity; time consuming; daily life disturbance	Needs based adjustment	Not reported
Gefen et al.^45^	Study-developed, pilot testing	Treatment/support	Treatment plan	None	Not reported	Not reported	Not reported
Grodon et al.^46^	Publicly available	Treatment/support; information sharing/communication	Interactive prompts; treatment plan; upload data	Reminder	Technical difficulties; technology availability; digital literacy; privacy and confidentiality; preference for in-person; lack of motivation	Ease of use; motivation; reminders; engagement with active features of the app	Added value to treatment, dependent on the professional role in demonstrating/explaining app
Hardy et al.^47^	Publicly available	Treatment/support	Treatment plan	None	Not reported	Usage pattern	Patient health condition impacted completion of components
Holyfield, Caron, Drager et al.^48^	Study-developed, under development	Treatment/support	Prompts; treatment plan	Bluetooth technology	Not reported	Not reported	Not reported
Holyfield, Caron, Light et al.^49^	Publicly available	Treatment/support	Treatment plan	None	Access to use	Motivation	Not reported
Johnson et al.^50^	Study-developed, pilot testing	Treatment/support; information sharing/communication	Interactive prompts; treatment plan; upload data	None	Not reported	Fun; goal setting; engagement with active ingredient of the app	Not reported
Kahana et al.^51^	Publicly available	Treatment/support	Treatment plan	One-way messaging	Not reported	Not reported	No improvements
Lorusso et al.^52^	Study-developed, pilot testing	Treatment/support	Prompts; treatment plan	None	Not reported	Accessibility features; ease of use; engaging graphics	Preference for activities that are engaging and active, with direct result reporting
Macdonald et al.^53^	Study-developed	Treatment/support	Prompts; treatment plan; upload data	None	Not reported	Not reported	Not reported
McLeod et al.^54^	Publicly available	Treatment/support; information sharing/communication	Interactive prompts; treatment plan; upload data	Electronic diary; Bluetooth technology	Parent involvement	Animations; rewards/points; parental engagement	Increased motivation
Memarian et al.^55^	Study-developed	Treatment/support; health promotion/prevention; information sharing/communication	Prompts; treatment plan	None	Not reported	Reduced burden	Not reported
Parnandi et al.^56^	Study-developed, pilot testing	Treatment/support	Prompts; treatment plan	None	Familiarity/novelty	Gamification; engaging graphics	Not reported
Patton et al.^57^	Study-developed, pilot testing	Health promotion; treatment/support	Interactive prompts	Electronic diary	Not reported	Parental engagement; app content	Not reported
Peterson et al.^58^	Publicly available	Treatment/support	Prompts; treatment plan; upload data	One-way messaging	Technological difficulties	Not reported	Not reported
Pramana et al.^59^	Study-developed, pilot testing	Treatment/support; health promotion/prevention; information sharing/communication	Prompts; treatment plan; two-way messaging; upload data	Electronic diary; reminders	Not reported	Not reported	Not reported
Pramana et al.^60^	Study-developed, pilot testing	Treatment/support; health promotion/prevention; information sharing/communication	Prompts; treatment plan; two-way messaging; upload data	None	Temperament	Engaging graphics; ease of use; usage pattern; motivation	Not reported
Pretlow et al.^61^	Study-developed, prototype	health promotion; treatment/support	Interactive prompts; Two-way messaging; upload data	One-way SMS and Messaging; Electronic diary; Bluetooth technology	Discomfort with peer support	Self-monitoring; mentor support; motivation	Not reported
Roca et al.^62^	Study-developed, pilot testing	Treatment/support	Prompts; treatment plan	None	Not reported	Not reported	Performed more exercises; well accepted; increased motivation compared to traditional treatment
Simmons et al.^63^	Study-developed, pilot testing	Treatment/support	Prompts; treatment plan; upload data	None	Not reported	Not reported	Not reported
Sreekumar et al.^64^	Publicly available	Treatment/support	Prompts; treatment plan	None	Not reported	Not reported	Not reported
Turner et al.^65^	Proprietary	Treatment/support	Treatment plan	Bluetooth technology; reminders	Temperament	Motivation; gamification; developmental	Not reported
Utepbayeva et al.^66^	Publicly available, pilot testing	Treatment/support	Interactive prompts; treatment plan; CDSS	Electronic diary	Privacy and confidentiality	Animations; rewards/points; games; engagement with active ingredients of the app	Increased motivation
Waddington et al.^67^	Publicly available	Treatment/support	Treatment plan	None	Not reported	Not reported	Not reported
Wade et al.^68^	Study-developed, pilot testing	Treatment/support	Prompts; treatment plan	Reminders	Familiarity/novelty; technological difficulties	Not reported	Not reported
Warmbier et al.^69^	Publicly available	Treatment/support; information sharing/communication	Interactive prompts; upload data	None	Not reported	Strategies for engagement; motivation; targeting, motivating and meaningful vocabulary; simple interactions	Increased motivation
Webb et al.^70^	Study-developed, pilot testing	Treatment/support; health promotion/prevention; information sharing/communication	Prompts; treatment plan	Electronic diary	Not reported	Not reported	Not reported
Worthen-Chaudhari et al.^71^	Study-developed, pilot testing	Treatment/support; health promotion/prevention	Two-way messaging; upload data	Electronic diary	Access to use	Not reported	Not reported
Yuan et al.^72^	Publicly available	Treatment/support	Prompts; treatment plan	None	Not reported	Not reported	Not 202reported
Zapata et al.^73^	Publicly available	Treatment/support	Prompts; treatment plan	None	Technological difficulties; access to use	Notifications; ease of use; previous experiences with apps/devices	Using an app for tracking exercise adherence was a barrier, not a supportive measure

*Note*.^1^App feature categories according to Donevant and colleagues.^20^

Small sample sizes were reported across several studies, with 19 studies (42%) having less than 10 participants. While most studies used only one test group, many studies incorporated different groups of participants, with a range between 1 to 3 groups, and only five (11%) studies reported using normalization groups. Nine studies (20%) incorporated parents or therapists as a targeted group. Of the controlled trials included, 10 compared mobile apps to non-app interventions, one compared between apps and 1 compared between both apps and other non-app interventions.

All studies provided details surrounding client/family access to technology, including providing technology as part of the study, technology owned by participants or both. Most of the studies provided a study-owned device to participants (*n* = 25, 55.5%), followed by participant/family-owned devices (*n* = 15, 33.3%), and a mix of study-owned and family-owned (*n* = 5, 11.1%). Devices that were used to enable mHealth during the interventions included tablet-only (*n* = 29, 64%), smartphones-only (*n* = 22, 49%), and were not specified (*n* = 6, 913%), with 12 studies (27%) using apps that were supported across multiple device types. A variety of operating systems were used to support the mHealth apps, with the majority using iOS (*n* = 24, 53.3%), android (*n* = 16, 35.5%), a few studies used both android and iOS (*n* = 9, 20%), and four studies (8.9%) did not report the compatible operating system(s) used.

mHealth app availability was also reported by a number of studies. The apps were reported to be study developed (*n* = 23, 51.1%), or publicly available (*n* = 15, 33.3%). The remaining seven studies (15.6%) did not provide information regarding what stage of development the app was. Only one study mentioned that the app was previously publicly available and then modified for use in their study. Studies were found to use one or more apps to enable mHealth; most of the studies reported using only one app (*n* = 32, 71%), followed by 2 – 4 apps (*n* = 9, 20%), and fewer used more than five (*n* = 4, 9%).

One characteristic of mHealth apps is the ability to pair with secondary technologies, which may include fitness trackers or geographic tagging. In one study, Near Field Communication (NFC) tags were attached to plastic animal toys for identification purposes, using electromagnetic induction between two loop antennae operating within an unlicensed radio frequency band.^52^ This technology enables NFC-enabled devices, such as a smartphone or tablet, to read information stored within the NFC tag (e.g., a zebra figurine would have an NFC tag coded with “Zebra”). A second study used Bluetooth to detect beacons to map out geographical locations.^37^ Finally, a third study used wrist-worn fitness bands that detected movement.^65^

### Participant characteristics

Studies were inconsistent in reporting participant ages, with one study (3%) not reporting any specific details on pediatric ages, with the remaining studies reporting the mean (*M*) ages (*n* = 3, 7%), age ranges (*n* = 7, 16%), individual ages (*n* = 10, 22%), or using a combination of reporting methods: *M* and range (*n* = 7, 16%), *M*, *SD*, and range (*n* = 13, 29%), and *M* and *SD* (*n* = 4, 9%). Using this information, we estimate that across all studies, participant ages ranged from 2.9 to 18 years, with a mean participant age of 10.44 years (*SD* = 3.97).

One goal of this scoping review was to examine if studies reported on child-specific criteria for using mHealth. Many studies reported study inclusion criteria according to participant ability to interact appropriately with mHealth tools and encompassed the child’s abilities and/or the absence of specific limitations (Table 4). A total of 23 studies did not report inclusion or exclusion criteria specific to mHealth use.

**Table 4.** mHealth-Specific Inclusion Criteria (N = 45).

Inclusion criterion	Count (*n*)
Child abilities	
Appropriate attention span	2
Communication ability*	3
Detect vibratory cue	1
Literacy	2
Motor control	4
Recognition of photos of people or objects	2
Use app	1
Use smartphone	1
Use symbolic communication (AAC)	2
Use social media sites to watch videos (Teacher reported)	1
Absence of	​
Auditory limitations*	4
Cognitive limitations	1
Developmental disorder*	1
Language barrier	6
Neurobehavioural disorder*	1
Physical limitations	1
Serious co-morbid medical condition affecting brain function and/or behaviour	1
Vision limitations	5
Child existing diagnoses	​
Apraxia of speech	1
Dysarthria	1
High Body Mass Index (BMI)	1
Speech delay	1
Fluency disorder	1
Phonological disorder	1
Articulation disorder	1
Hearing impairment	1
Diabetes	1

*Note*.*Includes studies where the parent self-indicated child’s abilities.

In 26 (58%) of the studies, the children used the apps independently without support for different sessions, while parents and/or therapists assisted children in using the apps in 20 (44%) of the studies. Five (11%) studies involved both independent child use and support from therapists and parents. The apps were used by children in their private therapy sessions in 17 (38%) studies, joint sessions with therapists, parents or other children in 26 (58%) studies, with 3 (7%) studies having both joint and private therapy sessions. Four studies did not indicate whether apps were used in private or joint therapy sessions.

The studies focused on a wide array of childhood health conditions. The research team combined the reported health conditions into six categories: 1) speech and language, 2) hearing, 3) mental health, 4) physical health, 5) autism spectrum disorder (ASD), and 6) developmental disorders. For the studies addressing speech and language conditions (*n* = 13, 29%), a variety of conditions were incorporated, including: childhood apraxia of speech; developmental language disorder; complex communication needs; speech and sound disorders, including prosody; general speech and language impairments including specific language impairment, prosody, and rhotic errors; moderate intellectual disability; and nonverbal diagnoses. Fifteen studies (33%) incorporated physical health conditions, including brain tumours, traumatic brain injury, painful hyperkyphosis, fibromyalgia, obesity, concussion, hydrocephalus, familial dysautonomia, and poor visual-motor skills. Mental health was included in six studies (13%), focusing on anxiety disorder, memory disorders, trait rumination, selective mutism, and psychological distress. Five of the included studies (11%) focused on children with autism spectrum disorder. Developmental conditions, a total of four studies (9%), included Fragile X syndrome as well as multiple developmental disorders. Finally, two studies (4%) focused on hearing conditions.

### mHealth characteristics

In alignment with the app feature categories outlined by Donevant and colleagues (2018), the studies included in this review included mHealth apps that contained more than one app component. Approximately 51% of the studies (*n* = 23) included apps that combined both interactive and passive components (Figure 2). Most commonly, the apps featured components related to the child’s action/treatment plan or personalized goals (n = 41). Overall, apps were less likely to contain passive components, with no studies using only passive components. On average, apps included 2.4 components (ranging from 1 to 6).

**Figure 2.**
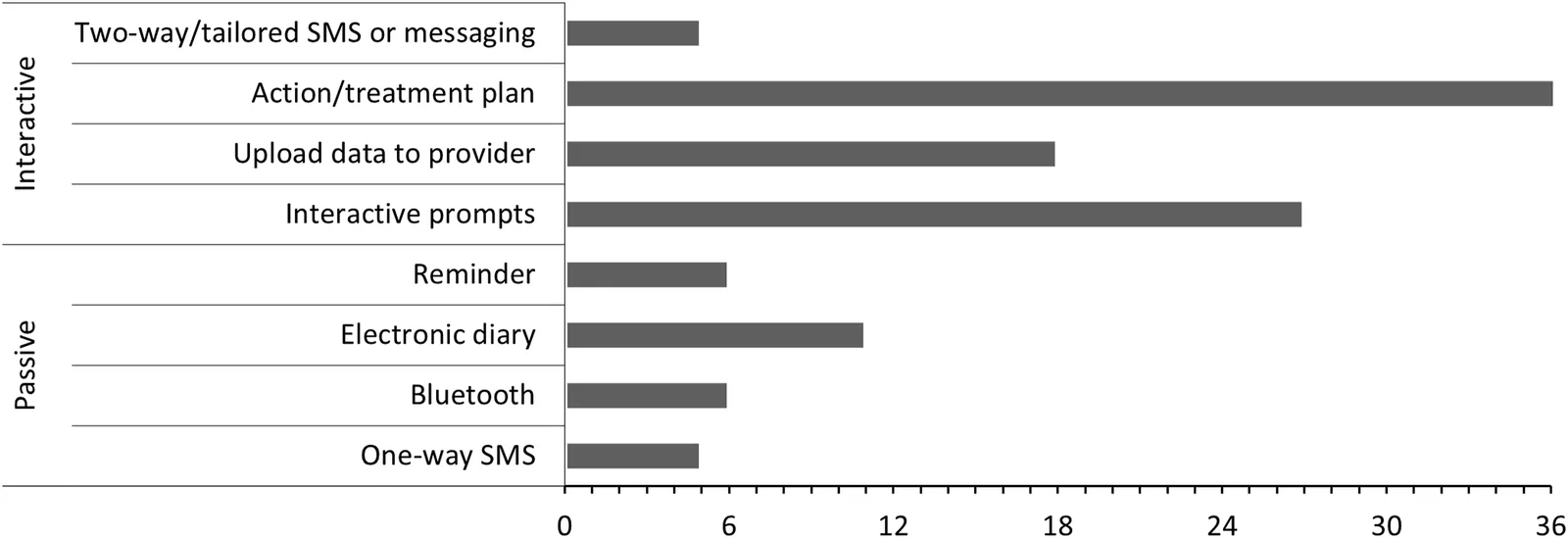
Interactive and passive app features.

A variety of locations and care scenarios were sampled across the included studies. The locations, or physical context, of the studies included home environments (*n* = 27, 60%); schools (*n* = 8, 18%); clinics (*n* = 15, 33%); other locations, including day care, work, or telepractice/video conference (*n* = 4, 9%); with seven studies occurring across multiple locations. Apps were involved in care during therapy sessions (*n* = 17, 38%), during daily life (*n* = 16, 36%), and during targeted behaviours or targeted situations (*n* = 21, 47%), with nine studies taking place during more than one scenario.

### App supports

Over half of the studies (*n* = 29, 64%) involved other individuals in supporting roles during app use by their child. These individuals included parents, caregivers, providers, members of the research team, or others (e.g., teachers, trained personnel, or coaches) mainly in the provision of support for younger children. The support roles, outlined in Table 5, included directing app use, coaching or other supports, set-up or installation, troubleshooting, training prior to the intervention, and viewing of results. A total of five studies incorporated parents or caregivers in one or more support roles. In addition to supportive roles, some studies reported providing pediatric participants with app-specific training or orientation prior to initiating mHealth use (*n =* 17, 47%).

**Table 5.** Type of support provided to the children during the care process (N = 45).

Category	Definition	Count (n)				
		Total	Parent	Provider	Research team	Other^1^
Directing app use	Directing app use as intervention, stating when and how often to use	38	1	19	16	2
Coaching	Providing guidance on use	26	3	13	5	5
Support during collaborative use	Prompted assistance and shared use of app	33	6	21	5	1
Set-up and installation support	Assisting with setting up mobile app and/or downloading software	8	1	4	3	0
Troubleshooting support	Assisting with technical challenges encountered	1	0	3	3	1
Training	Training on how to use app and in which situations provided prior to intervention	28	1	9	5	2
Viewing results	Post intervention, data upload and shared with provider or end user	28	1	15	10	2

*Note*.^1^Includes teachers, trained personnel, or coaches.

A total of 37 studies (82%) used feedback loops to deliver patient data to the clinician, with six studies not specifying the type of feedback. Out of these, 18 studies (40%) provided synchronous feedback, 13 (29%) provided asynchronous feedback, six (13%) provided a hybrid of synchronous, asynchronous, and/or in person feedback. A feedback loop, providing app feedback to the end-user, was used in 31 studies (69%), including synchronous feedback (*n* = 28, 62%), asynchronous feedback (*n* = 3, 7%); the remaining studies did not report whether data was shared back to the end user.

### Classification of evidence-level

An evaluation of evidence quality was conducted using the guidelines provided by Johns Hopkins Nursing.^27^ A total of 19 of the studies were randomized controlled trials or experimental studies with an RCT; 19 were either quasi-experimental or interventional studies with mixed methods. The remaining studies were qualitative (refer to Appendix D, Evidence Level).

## Discussion

This scoping review demonstrated that children’s engagement with mHealth applications is influenced by multiple design and functionality features that operate as both barriers and facilitators to sustained app use. When app engagement was examined as a study outcome, several factors were identified as facilitating children’s engagement with the application and/or their rehabilitative therapy. These included engaging visual design, ease of use, gamification elements, accessibility features, communication modalities, feedback mechanisms, usage patterns, motivational supports, opportunities for access, parental involvement, prior experience with similar apps or devices, notification systems, and developmental considerations. Barriers to child engagement were associated with developmental or temperament-related factors, the degree of familiarity or novelty of the application, cultural relevance and acceptance, inappropriate or challenging levels of gamification, limited access to use, technological difficulties, and general inconvenience. A persistent challenge for the design of future mHealth applications will be the need to integrate known facilitators while proactively addressing barriers to engagement. Notably, several factors, including gamification features, access to use, and developmental considerations, were identified as both facilitators and barriers, underscoring the complexity of designing universally engaging pediatric mHealth interventions. Achieving an optimal balance in gamification design, including one that appropriately challenges the user, offers meaningful rewards, and aligns with the user’s developmental level and skill capacity, will help advance effective mHealth app engagement.

mHealth applications are often free or low-cost; however, users can still encounter barriers such as limited device storage or mobility constraints, which may reduce access and/or engagement level. Developmental factors, especially age-related factors, further underscore the importance of designing apps that accommodate a wide developmental range and offer adjustable features tailored to a child’s specific abilities. Ease of use emerged as a key facilitator, whereas technological difficulties were consistently identified as barriers. Together, these findings underscore the need for design modifications that are sufficiently intuitive and developmentally appropriate for all intended users.

mHealth apps identified in this study were designed for direct, hands-on use by children, but also needed to integrate scenarios in which support persons (such as a parent or clinician) were needed to assist the child in real-world use. The results indicate that more than half of the children did not require any additional support to use the mHealth app in the real-world. These findings, related to children performing app-related tasks with little support, suggest an increased likelihood of future personal use; however, for many apps, the “users” extend beyond the child, underscoring additional design and development considerations. Parents and therapists are still needed to provide support to children; this was found across different age groups. mHealth apps can also be integrated in group sessions with other children, creating more avenues for their use. mHealth applications can facilitate interactive, individualized therapy activities involving direct child use and collaboration with caregivers and other professionals, when needed. A Table of clinical recommendations for pediatric mHealth app integration is included below (Table 6).

**Table 6.** Summary of recommendations for current clinical practice and future app development and design.

Clinical practice recommendations	Future app design considerations
• Assess child and family readiness to mHealth use. Consider developmental abilities, literacy, communication skills, and access to technology	• Design apps with developmentally appropriate interfaces tailored to pediatric abilities and needs
• Include caregivers, teachers, and other support personnel as active participants in app-supported rehabilitation and/or home practice as needed	• Integrate engaging features such as gamification, rewards, animations, and interactive graphics to enhance motivation
• Provide orientation, training, and troubleshooting support to children and families prior to app implementation	• Build accessibility features into app design to accommodate sensory, cognitive, motor, and language-related needs
• Select apps that are accessible, easy to use, and compatible with family-owned devices	• Develop apps that support flexible collaborative care models, including clinician, caregiver, and child interaction
• Integrate app use into individualized treatment goals and real-world daily routines to support carryover and adherence	• Incorporate integrated feedback loops and data-sharing systems to facilitate clinician monitoring and collaborative decision-making
• Use synchronous and/or asynchronous feedback to support monitoring, engagement, and therapeutic follow-up	• Improve interoperability across devices and operating systems to reduce technology-related barriers
• Consider clinician guidance and demonstration as part of implementation, as support from providers influenced engagement and perceived value	• Design apps with adaptable levels of support and independence based on child age, readiness, and caregiver involvement

Apps reported in the included articles were mainly compatible with either Android or iOS; only four articles reported an mHealth app compatible with both operating systems. System compatibility may be a potential barrier to app use, and healthcare providers may want to include loaner devices within their care setting, with their rehabilitation mHealth app already installed. The provision of these loaner devices has the potential to reduce barriers to technology access and/or compatibility, especially when accompanied by training to improve participants’ comfort with unfamiliar systems; this option was reported in many of the studies included in this review. Secondary technologies, such as fitness trackers, geographic tagging, and Bluetooth, were used in a handful of studies and should be further explored as they may be a means to encourage app engagement for pediatric end-users; however, their design should focus on facilitating app use and simplicity.

Included mHealth apps were either publicly available or study-developed, and in one case, a proprietary app was used.^65^ Most of the applications which were study-developed were being evaluated through pilot testing, relating to the novelty of mHealth applications as part of co-led rehabilitation interventions. Interactive features were more common than passive features (Table 3), with the interactive feature treatment plan found in the app design of almost all the included studies. This suggests the importance of inclusion of a treatment plan feature as part of a mHealth app design, as it serves as a quantifiable measure for personalized patient goals, which can be re-evaluated and modified as needed.^20^ Many of the studies did not have any passive features and only included interactive features relating to the importance of engaging the patient in their care process using a mHealth rehabilitative intervention. Given that many of the included studies had small sample sizes, evaluating the use of mHealth app(s) in a rehabilitation setting with a larger sample may yield more robust findings. Moreover, the use of different groups of participants and incorporating parents and therapists as part of the intervention can allow for the elucidation of various stakeholder perspectives.

The rapid integration of artificial intelligence (AI) will influence how mHealth is conceptualized and delivered in pediatric rehabilitation in the coming years. Many of the interactive features identified in this review, including personalized support, prompts, feedback, and communication, may change with the integration of AI, including generative AI or conversational AI tools. Mainstream, readily available AI tools may provide accessible, interactive, and adaptive support without requiring direct interaction with the intervention-specific app. These tools could create opportunities to enhance personalized pediatric rehabilitation care within existing platforms. Generative AI tools are not necessarily designed, validated, or governed for specific healthcare applications and may lack the clinical oversight and integration with care processes associated with purpose-built mHealth interventions.^74^ These considerations are important in pediatric rehabilitation, where careful attention should be paid to developmental appropriateness, caregiver involvement, clinical integration, including privacy considerations, and the accuracy of the AI-generated information. Future research should examine not only AI-enhanced mHealth applications but also how generative AI tools may complement, compete with, or alter the role of mHealth applications within collaborative rehabilitation care.

## Limitations

The majority of included studies (*n* = 39, 87%) originated from countries categorized as part of the Global North, including regions such as Canada and the United States, Australia and New Zealand, and the United Kingdom. The Global North is widely recognized as dominating knowledge production and dissemination, often shaping the standards and models that define publishable evidence.^75^ Findings from this scoping review highlight persistent inequities in global research representation, with only six articles (13%) including participants from the global South (China, India, Iran, Israel, Kazakhstan, and Slovenia). It is also important to note that this review was limited to English-language, peer-reviewed publications, which may have further contributed to the underrepresentation of research from non-English-speaking regions. As such, relevant studies published in other languages may have been excluded, potentially narrowing the global applicability of the findings.

The literature search was limited to health- and rehabilitation-focused databases to align with the clinical scope of the review and its emphasis on real-world implementation in pediatric rehabilitation. As a result, studies published in information science, engineering, or digital technology-focused databases and conference proceedings may not have been captured. Given the rapidly evolving nature of mHealth technologies, future reviews will benefit from updated searches and broader inclusion of digital and information science sources. Furthermore, rapidly emerging AI-enabled digital health approaches may not be adequately represented in the included evidence.

Reporting participant age presented a notable challenge, as age was described inconsistently across publications. Consequently, the mean participant age was calculated using the most complete information available from each study. The roles of individuals supporting the child’s app use also varied considerably, with some providing active assistance and others serving in more passive roles, patterns that may relate to the child’s age. However, the inconsistent reporting of age across studies limited the ability to identify meaningful commonalities or examine these relationships systematically.

Additionally, many of the included studies did not report on app quality and/or safety components. There are many tools available to evaluate app quality, privacy, and security for health-related apps; these have been developed for end-users, developers, and health professionals, including the Mobile Application Rating Scale or mHealth app usability questionnaire.^76,77^ Research teams should consider implementing and reporting on the use of these tools to better determine their appropriateness for clinical practice. No articles included in this review were identified or reported to have used an evaluation tool on the mHealth apps used in their research protocols. Emerging research supports the use of such tools to guide future app design and appropriately assess the clinical usability.^78–80^

## Conclusion

While many mHealth apps for pediatric allied health care are available on the market, the literature supporting their use in real-world, collaborative settings is limited. Despite the growing availability of digital tools, most studies included in this review were early-stage evaluations with small sample sizes, inconsistent reporting of participant age and characteristics, and limited descriptions of implementation contexts. Although mHealth interventions showed promise across diverse pediatric conditions, ranging from speech and language to mental health, physical rehabilitation, autism spectrum disorder, and developmental disorders, the overall evidence base remains insufficient to guide widespread clinical adoption.

Findings from this scoping review highlight that children’s engagement with mHealth tools is shaped by a complex interplay of design, usability, contextual, and developmental factors that can serve as both facilitators and barriers. Interactive app features, including treatment plan components, prompts, real-time communication, and feedback mechanisms, were most strongly associated with positive engagement, whereas factors such as technological difficulties, system incompatibility, limited device access, and developmental appropriateness challenges frequently reduced engagement. Importantly, several key factors such as gamification, access constraints, and developmental considerations operated as both facilitators and barriers, underscoring the complexity of designing mHealth apps that are universally engaging and developmentally attuned for pediatric users. Overall, this review illustrates the need for more rigorous, developmentally informed, and contextually grounded research evaluating pediatric mHealth tools within real-world rehabilitation settings. Greater attention to app quality and safety, standardized reporting, and the use of validated evaluation frameworks will be essential for advancing equitable, evidence-based digital care and strengthening the integration of mHealth within collaborative pediatric rehabilitation models.

## Supplemental material

Supplemental material - Child-focused mHealth apps used in collaborative, real-world interventions: A scoping review Supplemental material for Child-focused mHealth apps used in collaborative, real-world interventions: A scoping review by S. Pardal, R. O’Hagan, E. Abaya-Kotey, J. A. Schnittker and D. Glista in Digital health.

## Appendix

### List of abbreviations

AUD: Audiologist
OT: Occupational therapist
PCC: Person, Concept, Context
PRISMA: Preferred Reporting Items for Systematic reviews and Meta-Analyses
PT: Physiotherapist
SES: Socio-economic status
SLP: Speech Language Pathologist.
