# Supplementary material for: Child-focused mHealth apps used in collaborative, real-world interventions: A scoping review
Source: Digit Health. 2026 Sep 18;12:20552076261490207. doi: 10.1177/20552076261490207 (PMC13724959; doi:10.1177/20552076261490207)
Supplement: Supplementary file 1 [file sj-pdf-1-dhj-10.1177_20552076261490207.pdf]

Appendix A

Preferred Reporting Items for Systematic reviews and Meta-Analyses extension for Scoping Reviews (PRISMA-ScR) Checklist

SECTION	ITEM	PRISMA-ScR CHECKLIST ITEM	REPORTED ON PAGE #
**TITLE**			
Title	1	Identify the report as a scoping review.	1
**ABSTRACT**			
Structured summary	2	Provide a structured summary that includes (as applicable): background, objectives, eligibility criteria, sources of evidence, charting methods, results, and conclusions that relate to the review questions and objectives.	2
**INTRODUCTION**			
Rationale	3	Describe the rationale for the review in the context of what is already known. Explain why the review questions/objectives lend themselves to a scoping review approach.	5
Objectives	4	Provide an explicit statement of the questions and objectives being addressed with reference to their key elements (e.g., population or participants, concepts, and context) or other relevant key elements used to conceptualize the review questions and/or objectives.	6
**METHODS**			
Protocol and registration	5	Indicate whether a review protocol exists; state if and where it can be accessed (e.g., a Web address); and if available, provide registration information, including the registration number.	8
Eligibility criteria	6	Specify characteristics of the sources of evidence used as eligibility criteria (e.g., years considered, language, and publication status), and provide a rationale.	8
Information sources*	7	Describe all information sources in the search (e.g., databases with dates of coverage and contact with authors to identify additional sources), as well as the date the most recent search was executed.	9
Search	8	Present the full electronic search strategy for at least 1 database, including any limits used, such that it could be repeated.	9
Selection of sources of evidence†	9	State the process for selecting sources of evidence (i.e., screening and eligibility) included in the scoping review.	10
Data charting process‡	10	Describe the methods of charting data from the included sources of evidence (e.g., calibrated forms or forms that have been tested by the team before their use, and whether data charting was done independently or in duplicate) and any processes for obtaining and confirming data from investigators.	10
Data items	11	List and define all variables for which data were sought and any assumptions and simplifications made.	44
Critical appraisal of individual sources of evidence§	12	If done, provide a rationale for conducting a critical appraisal of included sources of evidence; describe	N/A

SECTION	ITEM	PRISMA-ScR CHECKLIST ITEM	REPORTED ON PAGE #
		the methods used and how this information was used in any data synthesis (if appropriate).	
Synthesis of results	13	Describe the methods of handling and summarizing the data that were charted.	11
**RESULTS**			
Selection of sources of evidence	14	Give numbers of sources of evidence screened, assessed for eligibility, and included in the review, with reasons for exclusions at each stage, ideally using a flow diagram.	12
Characteristics of sources of evidence	15	For each source of evidence, present characteristics for which data were charted and provide the citations.	13
Critical appraisal within sources of evidence	16	If done, present data on critical appraisal of included sources of evidence (see item 12).	N/A
Results of individual sources of evidence	17	For each included source of evidence, present the relevant data that were charted that relate to the review questions and objectives.	15
Synthesis of results	18	Summarize and/or present the charting results as they relate to the review questions and objectives.	20
**DISCUSSION**			
Summary of evidence	19	Summarize the main results (including an overview of concepts, themes, and types of evidence available), link to the review questions and objectives, and consider the relevance to key groups.	26
Limitations	20	Discuss the limitations of the scoping review process.	28
Conclusions	21	Provide a general interpretation of the results with respect to the review questions and objectives, as well as potential implications and/or next steps.	29
**FUNDING**			
Funding	22	Describe sources of funding for the included sources of evidence, as well as sources of funding for the scoping review. Describe the role of the funders of the scoping review.	31

JBI = Joanna Briggs Institute; PRISMA-ScR = Preferred Reporting Items for Systematic reviews and Meta-Analyses extension for Scoping Reviews.

* Where *sources of evidence* (see second footnote) are compiled from, such as bibliographic databases, social media platforms, and Web sites.

† A more inclusive/heterogeneous term used to account for the different types of evidence or data sources (e.g., quantitative and/or qualitative research, expert opinion, and policy documents) that may be eligible in a scoping review as opposed to only studies. This is not to be confused with *information sources* (see first footnote).

‡ The frameworks by Arksey and O'Malley (6) and Levac and colleagues (7) and the JBI guidance (4, 5) refer to the process of data extraction in a scoping review as data charting.

§ The process of systematically examining research evidence to assess its validity, results, and relevance before using it to inform a decision. This term is used for items 12 and 16 instead of "risk of bias" (which is more applicable to systematic reviews of interventions) to include and acknowledge the various sources of evidence that may be used in a scoping review (e.g., quantitative and/or qualitative research, expert opinion, and policy document).

Appendix B

Final Search Strategies

The following search strategies were used and the articles obtained were uploaded to Covidence for screening.

Database: Ovid MEDLINE(R) ALL <1946 to November 25, 2024>

1. Pediatrics/ (57870)
2. (pediatric* or paediatric*).tw,kf. (467774)
3. Child/ (1911754)
4. child*.tw,kf. (1674610)
5. Child, Preschool/ (989385)
6. Adolescent/ (2214888)
7. adolescent*.tw,kf. (326227)
8. teen*.tw,kf. (35715)
9. 1 or 2 or 3 or 4 or 5 or 6 or 7 or 8 (3989911)
10. Mobile Applications/ (11430)
11. "mobile app*".tw,kf. (10076)
12. Telemedicine/ (37289)
13. (mHealth* or m-Health* or "mobile health*").tw,kf. (14330)
14. "mobile treatment*".tw,kf. (50)
15. "mobile intervention*".tw,kf. (318)
16. "smartphone app*".tw,kf. (6330)
17. "connected health*".tw,kf. (336)
18. 10 or 11 or 12 or 13 or 14 or 15 or 16 or 17 (61630)
19. Audiology/ (2335)
20. Audiologists/ (214)
21. audiolog*.tw,kf. (12635)
22. Speech-Language Pathology/ (3679)
23. ((speech or language) adj3 (therap* or patholog*)).tw,kf. (13771)
24. "communication sciences and disorders".tw,kf. (131)
25. (communication adj3 disorder*).tw,kf. (2545)
26. Occupational Therapy/ (14841)
27. "occupational therap*".tw,kf. (18279)
28. Physical Therapists/ (3115)
29. ("physical therap*" or physiotherap*).tw,kf. (63987)
30. prosthet*.tw,kf. (64099)
31. orthot*.tw,kf. (40515)
32. Psychiatric Rehabilitation/ or "Physical and Rehabilitation Medicine"/ or Rehabilitation Nursing/ or Rehabilitation/ (23580)
33. Psychology, Clinical/ (3269)
34. "clinical psychol*".tw,kf. (5230)
35. "physical medicine doctor*".tw,kf. (3)
36. (rehab* adj3 (doctor* or nurse*)).tw,kf. (1143)

37. (rehab* adj3 (professional* or "science professional*" or science*)).tw,kf. (2981)
38. rehabilitation/ or "activities of daily living"/ or animal assisted therapy/ or art therapy/ or bibliotherapy/ or cardiac rehabilitation/ or "correction of hearing impairment"/ or dance therapy/ or early ambulation/ or exercise therapy/ or music therapy/ or neurological rehabilitation/ or occupational therapy/ or recreation therapy/ or "rehabilitation of speech and language disorders"/ or rehabilitation, vocational/ or telerehabilitation/ (174381)
39. 19 or 20 or 21 or 22 or 23 or 24 or 25 or 26 or 27 or 28 or 29 or 30 or 31 or 32 or 33 or 34 or 35 or 36 or 37 or 38 (376409)
40. 9 and 18 and 39 (329)

Appendix C

Data Extraction Tool

Factor/theme	Extraction details
Study objectives	Primary and secondary research goals
Country of origin	Location of participants or location of affiliated authors
Study design	Type of data collection method used
Pilot or efficacy study	Yes/no, if yes, provide details
Were there multiple components in the care process?	Provide details (e.g., paper-based diary, physical activity/activity monitor, video conference check-in)
What was the real-world, physical context(s) of App use?	Yes/no, if yes, provide details
Care scenario	Provide details
Rehabilitation Professionals Involved	Select from list or provide details
Who specifically delivered the intervention?	Provide details
Childhood health condition targeted in intervention	(e.g., Autism, hearing loss)
Number of participants per cohort	Describe all involved in intervention: children, parents, providers, etc. Include details if there are multiple study groups (e.g., normal versus treatment)
Pediatric participants age range	Provide details - Mean, Range, SD, etc.
What were the inclusion criteria for selecting pediatric participants, specific to mHealth use?	Provide details
What were the interactive features of the app?	Select from: interactive prompts, upload data to provider, action/treatment plan or personalized goals, two-way or tailored SMS or messaging
Did the app include any of the following passive features?	Select from: one-way SMS, Bluetooth, electronic diary, reminders
Did another person support app use? (caregiver, peer)	Yes/no, if yes, include details on their role in app use/shared use
Describe the provider and/or collaborative care	Provide details (e.g. the provider role in dictating app use, use together during session, or viewing results after intervention)
App-specific Training/ Orientation Provided	Yes/no, if yes, describe type and timeline
Access to technology	Provide details
Name of App(s)	Provide the name of the app(s) included in the study and any details around age-specifics
How many apps were used in the study?	n
What device was used to support the App(s)?	Provide details
What operating system(s) were used with the App(s)? Were there any comparisons made?	iOS, Android, both, or other. Provide details when possible.
App source	Include details (e.g., publicly available or study-developed)
App stage of development	Include details (e.g., Study prototype, testing, feasibility, etc.)
App(s) objective	Provide details if different from study objective or state if targeting one component of study objective
Is the App paired to other external devices?	Yes/No, if yes, provide details (e.g., smart watch, activity tracker, scale)

Factor/theme	Extraction details
App interface details	Include relevant details to each component (e.g., learning modules, gamification, storytelling)
Design features of App	Include details (e.g. personalization, accessibility, security/passcode, notifications or push prompts, chat).
Frequency of App use	Provide details (e.g., 5x a week for 15 minutes)
Feedback delivery model to clinician/research team	Real-time, asynchronous, or none
If feedback is provided to clinician/research team, what is shared/accessed?	Yes/No, if yes, provide details.
Did the app integrate A.I. components?	Yes/No, if yes, provide details.
Quality assessment and results	Yes/No, if yes, provide details.
Safety assessment and results	Yes/No, if yes, provide details.
User-engagement assessment and results	Yes/No, if yes, provide details.
Child's baseline app acceptance and/or comfort with use (pre-intervention)	Provide details
Child's post-intervention app comfort or acceptance	Provide details
Child-targeted usability of the app	Provide details on how the child was able to interact with or use the app
Clinician acceptance/assessment of app	Provide details
App engagement barriers	Provide details
App engagement facilitators	Provide details
General Outcomes	Provide details
Did app use influence the child(ren)'s engagement in care?	Yes/No, if yes, provide details.
Details of interesting/relevant case studies	Provide details
Did this study identify any candidacy factors, please list:	Yes/No, if yes, provide details.
Future directions/real-world applicability/clinical applicability/author suggestions	Provide details

Appendix D

Summary of Included Articles

Citation	Study Origin	Evidence level	Type of app assessment[1]	Rehabilitation professional[2]	Total participants: childhood health condition	Participant ages (range, years)
Ahmed et al. (2018)	USA, Australia, Qatar	1: RCT	Feasibility	S-LP, research team	10: Childhood apraxia of speech (CAS)<br>6: Typical development	6 – 11
Babb et al. (2021)	USA	2: Experimental study with mixed method design	None	S-LP, psychologist, research team	4: Autism spectrum disorder (ASD) and complex communication needs	16 – 18
Badesha et al. (2023)	Great Britain	3: Qualitative study	None	Psychologist, mental health practitioner	5: Psychological distress	15 – 17
Bent et al2024)	USA	2: Quasi experimental study	Feasibility, acceptability	PT, Physical Medicine Doctor, Certified athletic trainer	15: Overweight with spina bifida	12 – 18
Brouwer et al. (2017)	USA	2: Experimental study with mixed method study design	None	S-LP, AUD	2: Normal hearing<br>2: Hearing loss and utilized cochlear implant	3 – 4
Bunnell et al. (2018)	USA	1: RCT	None	Psychologist	15: Selective mutism	5 – 17
Caron et al. (2018)	USA	2: Experimental study with mixed method design	None	S-LP	5: ASD and complex communication needs	6 – 14
Carson (2020)	Australia	2: Quasi experimental study	None	S-LP	24: Developmental language disorder (DLD)	4 – 5
Chan et al. (2020)	China	2: Experimental study with mixed method design	None	S-LP	6: Moderate intellectual disability/nonverbal diagnosis	Not reported
Chung K. & Chung E. (2023)	Korea	1: RCT	None	Psychologist	67: ASD	7 – 15
Coutinho et al. (2017)	Canada	1: RCT	None	OT	20: Children with poor visual-motor skills and special needs	6.4[3]
De-Jongh González et al. (2022)	Canada	1: RCT	Usability	Psychologist, rehabilitation professional	214: Overweight and obesity	10 – 17

de la Vega et al. (2018)	USA, Spain	3: Qualitative study	Acceptability, feasibility, satisfaction, usability	Psychologist	25: Fibromyalgia or chronic widespread pain	13 – 24
Diez-Juan et al. (2014)	USA, Spain	1: RCT	None	Psychologist, rehabilitation professional	4: Fragile X Syndrome	3 – 10
Gačnik et al. (2018)	Russia, Slovenia	2: Experimental study with mixed method design	Usability	S-LP	82: Speech sound disorders	3 – 12
Gazibegovic et al (2024)	Germany	2. Experimental study with mixed design	Feasibility, acceptability	AUD, rehabilitation professional	16: Hearing loss	8 – 12
Gefen et al. (2015)	Israel	3: Qualitative study	Feasibility	PT	3: Familial dysautonomia with gait and stability problems	11 – 22
Grodon et al (2024)	UK	2. Experimental study with mixed design	Feasibility, acceptability	PT	8: Musculoskeletal (MSK) conditions	16 – 18
Hardy et al. (2021)	USA	1: RCT	Feasibility	Psychologist	47: Sickle cell disease with short-term memory or working memory difficulties	10.4[3]
Holyfield, Caron, Drager, et al. (2019)	USA	2: Experimental study with mixed method design	None	S-LP	5: Beginning communicators	9 – 18
Holyfield, Caron, Light, et al. (2019)	USA	2: Experimental study with mixed method design	None	S-LP	3: Children with multiple disabilities	5 – 12
Johnson et al (2024)	Australia, New Zealand	2: Experimental study with a mixed method design	Feasibility, acceptability	S-LP, OT, PT	8: Children with neurodisabilities	6 – 11
Kahana et al. (2022)	Israel	3: Qualitative study	None	PT	95: Children with overweight and obesity	9[3]
Lorusso et al. (2018)	Italy	3: Qualitative study	Satisfaction, usability	S-LP	14: Various specific language impairment (expressive only, receptive & expressive, prominently phonological, prominently lexical-syntactic	4 – 6
Macdonald et al. (2022)	Canada	2: Quasi experimental study	None	Psychologist	16: Typical development; 16: ASD, ASD + hyperlexia	3 – 6
McLeod et al (2023)	Australia	3: Qualitative study	Feasibility, acceptability	S-LP	45: Speech Sound disorder	4 – 12
Memarian et al. (2022)	Australia, Iran	1: RCT	Feasibility	Psychologist	46: Overweight or obesity	9.3[3]

Parnandi et al. (2015)	Australia, Qatar, USA	2: Experimental study with mixed method design	Feasibility, satisfaction, usability	S-LP	7: CAS	4 – 10
Patton et al (2024)	USA	1: RCT	Feasibility, acceptability	Psychologist, Physical Medicine Doctor	10: Type 1 Diabetes	8 – 12
Peterson et al. (2022)	USA	1: RCT	None	S-LP	4: Residual rhotic errors affecting American English	9 – 10
Pramana et al. (2014)	USA	2: Quasi experimental study	Acceptability, feasibility, satisfaction, usability	Psychologist	9: Anxiety	9 – 14
Pramana et al. (2018)	USA	2: Quasi experimental study	Acceptability	Psychologist	35: Generalized anxiety disorder, social anxiety disorder, and/or separation anxiety disorder	9 – 14
Pretlow et al (2015)	USA, Australia	2: Quasi experimental study	Feasibility, acceptability	Physical Medicine Doctor, Rehabilitation Nurse	27: Overweight/obesity	10 – 18
Rocha et al. (2019)	Portugal	2: Quasi experimental study	Satisfaction, usability	S-LP	20: Typical development; 15: Speech and language impairments	7 – 10
Simmons et al. (2016)	USA	3: Qualitative study	Acceptability, feasibility	S-LP	40: Prosodic deficits in children with ASD and other communication impairments	9.6[3]
Sreekumar S. et al. (2020)	India	3: Qualitative study	None	S-LP	1: Complex communication needs	7
Turner et al. (2023)	Great Britain	2: Experimental study with mixed method design	Acceptability, feasibility	OT, PT, psychologist, research team	17: Unilateral cerebral palsy; 16: paired peers	8 – 17
Utepbayeva et al (2024)	Kazakhstan	1: RCT	Feasibility, acceptability	S-LP	170: Speech disorders	3 – 5
Waddington et al. (2014)	USA, New Zealand, Italy, Austria	2: Experimental study with mixed method design	None	Psychologist	3: ASD	7 – 10
Wade et al. (2018)	USA	2: Quasi experimental study	Acceptability, feasibility, satisfaction, usability	OT, physical medicine doctor, college student coach	6: Brain tumour; 9: Traumatic brain injury	14 – 22

Warmbier et al (2023)	Poland	3: Qualitative study		S-LP	1: Autism, neurodevelopment disorder, speech development delay	4
Webb et al. (2022)	USA	1: RCT	None	Psychologist	152: Trait rumination	12 – 15
Worthen-Chaudhari et al. (2017)	USA, Canada	2: Quasi experimental study	Feasibility, satisfaction, usability	Rehabilitation doctor	36: Concussion	13 – 18
Yuan et al. (2016)	USA	2: Quasi experimental study	None	OT	5: Surgically treated hydrocephalus	6 – 9
Zapata et al. (2018)	USA	2: Quasi experimental study	Acceptability, satisfaction, usability	PT	14: Painful hyperkyphosis	15.3[3]

Note. [1]Denotes the assessment completed according to User Engagement Indicator (UEI) categories (Ng et al., 2019). [2]Rehabilitation professionals have been abbreviated (S-LP: Speech Language Pathologist, OT: Occupational Therapist, PT: Physiotherapist, AUD: audiologist. [3]Ages were reported as a Mean value.
